# Supplementary material for: Rolling-Translated circRUNX2.2 Promotes Lymphoma Cell Proliferation and Cycle Transition in Marek’s Disease Model
Source: Int J Mol Sci. 2024 Oct 25;25(21):11486. doi: 10.3390/ijms252111486 (PMC11545863; doi:10.3390/ijms252111486)
Supplement: Supplementary file 1 [file ijms-25-11486-s001.zip › Supplementary Figure S1.pdf]

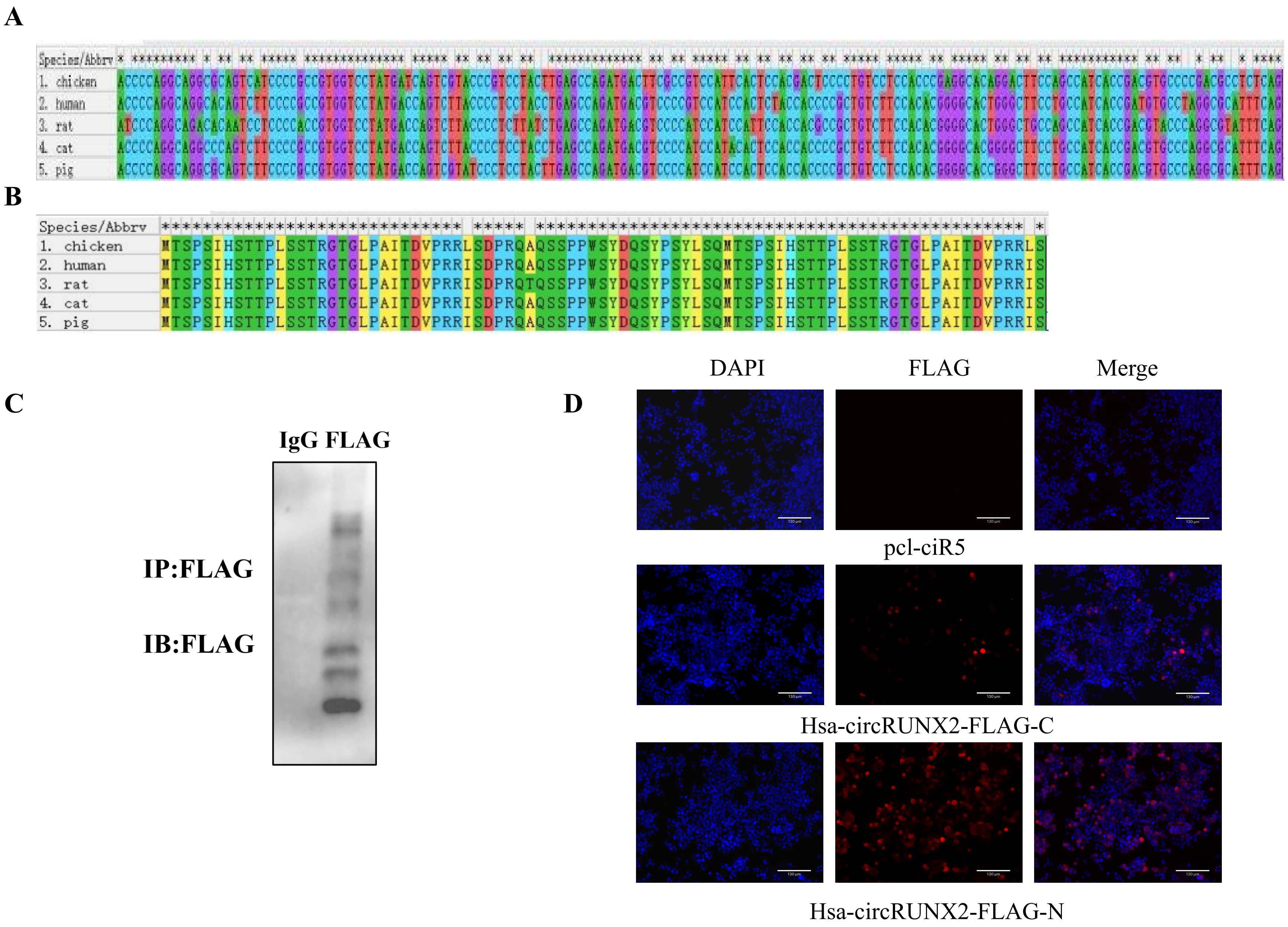
A
Species/Abbrv
1. chicken
2. human
3. rat
4. cat
5. pig
B
Species/Abbrv
1. chicken MTSPSIHSTTPLSSTRGTGLPAITDVPRRLSDPRQAQSSPPWSYDQSYPSYLSQMTSPSIHSTTPLSSTRGTGLPAITDVPRRLS
2. human MTSPSIHSTTPLSSTRGTGLPAITDVPRRISDPRQAQSSPPWSYDQSYPSYLSQMTSPSIHSTTPLSSTRGTGLPAITDVPRRIS
3. rat MTSPSIHSTTPLSSTRGTGLPAITDVPRRISDPRQTQSSPPWSYDQSYPSYLSQMTSPSIHSTTPLSSTRGTGLPAITDVPRRIS
4. cat MTSPSIHSTTPLSSTRGTGLPAITDVPRRISDPRQAQSSPPWSYDQSYPSYLSQMTSPSIHSTTPLSSTRGTGLPAITDVPRRIS
5. pig MTSPSIHSTTPLSSTRGTGLPAITDVPRRISDPRQAQSSPPWSYDQSYPSYLSQMTSPSIHSTTPLSSTRGTGLPAITDVPRRIS
C
IgG FLAG
IP:FLAG
IB:FLAG
D
DAPI
FLAG
Merge
pcl-ciR5
Hsa-circRUNX2-FLAG-C
Hsa-circRUNX2-FLAG-N
