# Supplementary material for: Rolling-Translated circRUNX2.2 Promotes Lymphoma Cell Proliferation and Cycle Transition in Marek’s Disease Model
Source: Int J Mol Sci. 2024 Oct 25;25(21):11486. doi: 10.3390/ijms252111486 (PMC11545863; doi:10.3390/ijms252111486)
Supplement: Supplementary file 1 [file ijms-25-11486-s001.zip › Supplementary Figure S3.pdf]

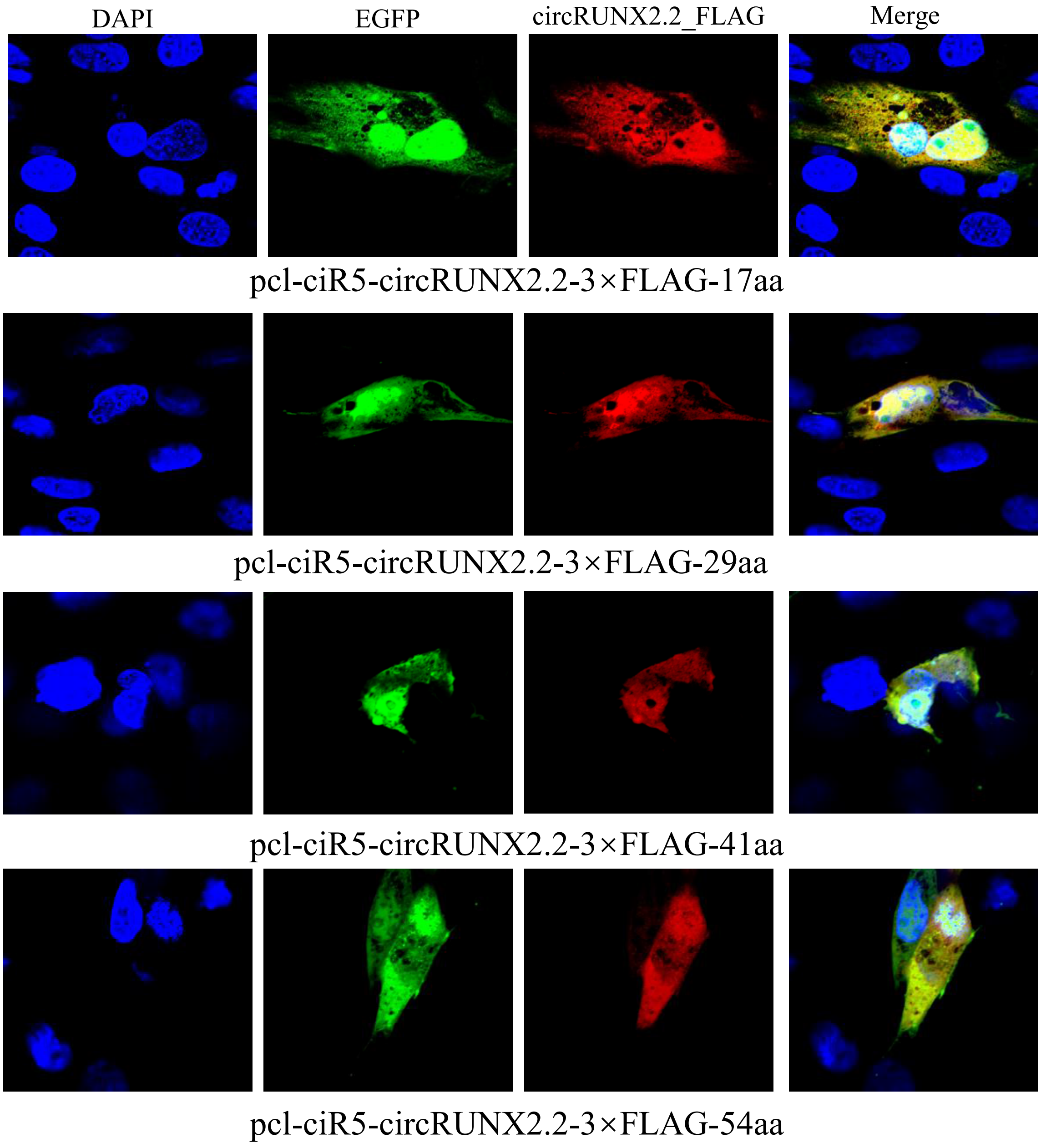
DAPI
EGFP
circRUNX2.2_FLAG
Merge
pcl-ciR5-circRUNX2.2-3×FLAG-17aa
pcl-ciR5-circRUNX2.2-3×FLAG-29aa
pcl-ciR5-circRUNX2.2-3×FLAG-41aa
pcl-ciR5-circRUNX2.2-3×FLAG-54aa
