# Supplementary material for: Rolling-Translated circRUNX2.2 Promotes Lymphoma Cell Proliferation and Cycle Transition in Marek’s Disease Model
Source: Int J Mol Sci. 2024 Oct 25;25(21):11486. doi: 10.3390/ijms252111486 (PMC11545863; doi:10.3390/ijms252111486)
Supplement: Supplementary file 1 [file ijms-25-11486-s001.zip › Supplementary information.pdf]

Supplementary information

Supplementary Table S1 Mass spectrometric results of circRUNX2.2-FLAG-interacting proteins

Supplementary Table S2 The information of primer and probe sequences used for this experiment.

Supplementary Table S3 The information of differentially expressed genes in cluster 2 and cluster 5.

Supplementary Figure S1 Conservativeness analysis of circRUNX2.2. A Homology comparison for nucleotide sequence of RUNX2 exon6 fragment encoding circRUNX2.2 with length of 162 nt among different species. B Homology comparison for predicted amino acid sequences of circRUNX2.2-encoded small peptides among different species. C Human circRUNX2.2 encoded small peptide validation. Human circRUNX2.2-FLAG vector was constructed and transferred into 293T cells, and FLAG antibody was used to detect circRUNX2.2-FLAG fusion protein. D Detection of peptide encoded by human circRUNX2.2 via IF assay. The ORF of human-derived circRUNX2.2 with N-terminal and C-terminal FLAG-fused vectors was constructed and transfected into 293T cells, circRUNX2.2-FLAG expression signals were detected by IF using FLAG antibody. FLAG: the red fluorescent, nucleus was stained with DAPI (blue).

Supplementary Figure S2 Subcellular localization of translation products of circRUNX2.2_RFP fusion vector. **A-C** The pcl-ciR5-RFP, pcl-cirR5-ΔATG and pcl-ciR5-circRUNX2.2-RFP-ΔATG vectors were transfected into DF1 cells for 48 h, separately. RFP expression was detected via fluorescence microscope. **D** The pcl-ciR5-circRUNX2.2-ΔATG-RFP-ΔATG vector which the ATG of the ORF on pcl-ciR5-circRUNX2.2-RFP-ΔATG was deleted was transfected into DF1 cells for 48 h. RFP expression was detected via fluorescence microscope. **E-H** The pcl-ciR5-circRUNX2.2-RFP-ΔATG-ΔTAA-17aa, pcl-ciR5-circRUNX2.2-RFP-ΔATG-ΔTAA-29aa, pcl-ciR5-circRUNX2.2-RFP-ΔATG-ΔTAA-41aa, and pcl-ciR5-circRUNX2.2-RFP-ΔATG-ΔTAA-54aa vectors were transfected into DF1 cells for 48 h, separately. RFP expression was detected via fluorescence microscope. Confocal laser microscopy was used to detect the distribution of red fluorescence. Scale bars = 10 μM.

Supplementary Figure S3 Subcellular localization of translation products of circRUNX2.2_

FLAG fusion vector. The pcl-ciR5-circRUNX2.2-3×FLAG-17aa, pcl-ciR5-circRUNX2.2-3×FLAG-29aa, pcl-ciR5-circRUNX2.2-3×FLAG-41aa, and pcl-ciR5-circRUNX2.2-3×FLAG-54aa vectors were transfected into DF1 cells for 48 h. Immunofluorescent staining with anti-FLAG were used to detect the distribution of fused translation products in cells. Scale bars = 10 μM.

Supplementary Figure S4 The effect of circRUNX2.2 on the expression profile of MSB1 cells. A Hotspot maps of differentially expressed genes. E: MSB1 cells were treated with pcl-ciR5, W: MSB1 cells were treated with circRUNX2.2, W: MSB1 cells were treated with circRUNX2.2-ΔATG. B Expression trend analysis of differentially expressed genes (DEGs). Upper panel: profiles ordered based on the p-value of number of genes assigned versus expected. Lower panel: profiles ordered based on the number of genes assigned. C-D Enrichment function analysis of 40 DEGs in cluster 2 for GO (c) and KEGG (d). E-F Enrichment function analysis of 77 DEGs in cluster 5 for GO (e) and KEGG (f).
