# Supplementary figures and images for: Rolling-Translated circRUNX2.2 Promotes Lymphoma Cell Proliferation and Cycle Transition in Marek’s Disease Model
Source: Int J Mol Sci. 2024 Oct 25;25(21):11486. doi: 10.3390/ijms252111486 (PMC11545863; doi:10.3390/ijms252111486)

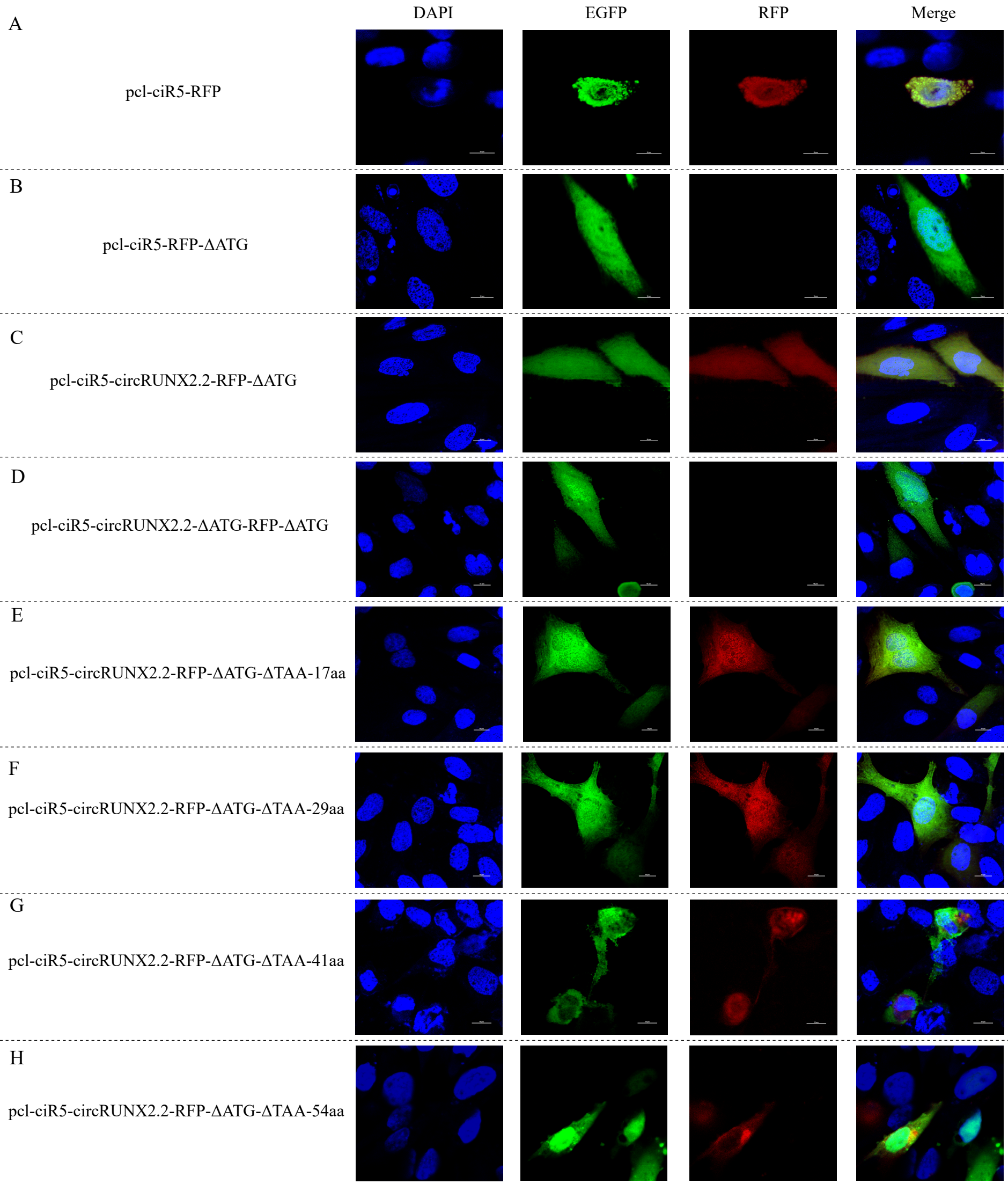

Supplement: Supplementary file 1 [file ijms-25-11486-s001.zip › Supplementary Figure S2.pdf]

A

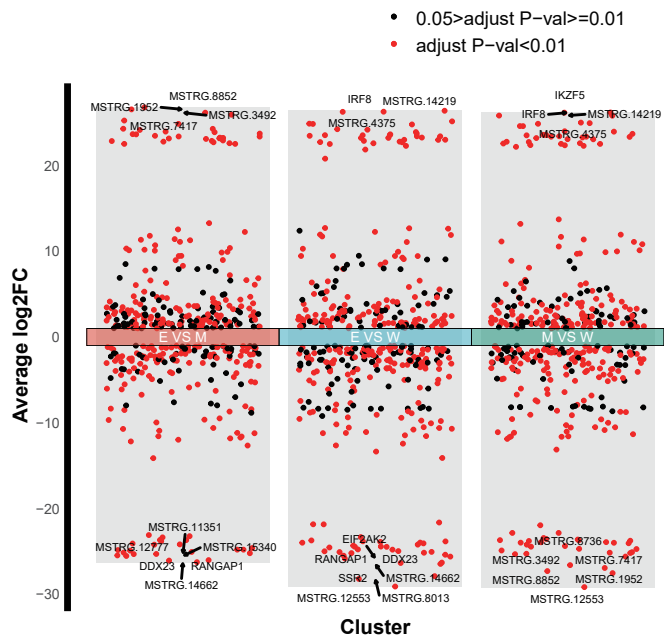

B

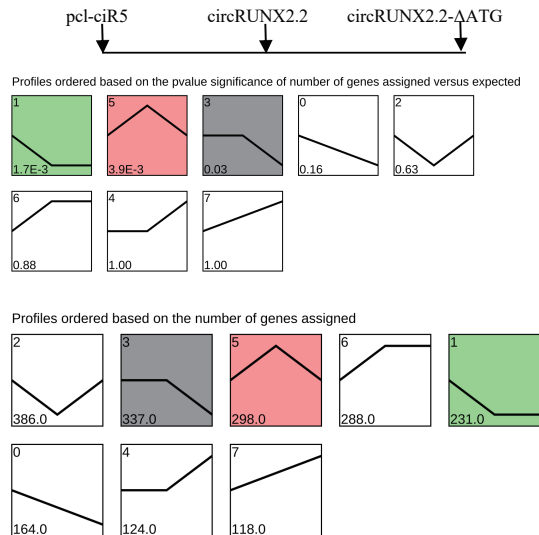

C

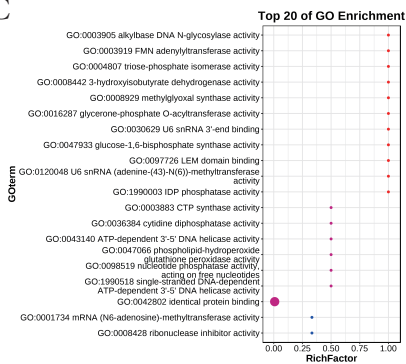

D

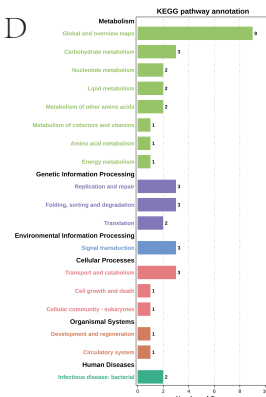

E

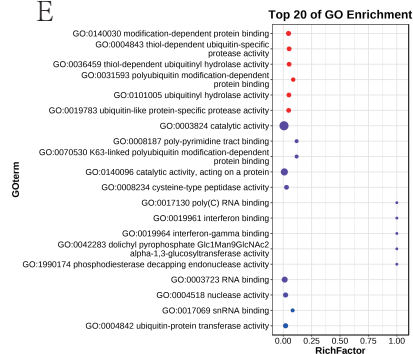

F

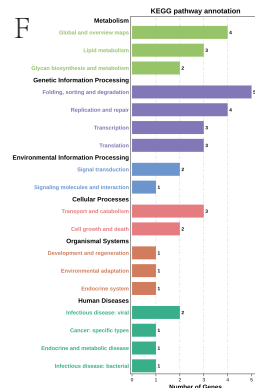

Supplement: Supplementary file 1 [file ijms-25-11486-s001.zip › Supplementary Figure S4.pdf]
